# Pathogenicity Islands Distribution in Non-O157 Shiga Toxin-Producing *Escherichia coli* (STEC)

**DOI:** 10.3390/genes9020081

**Published:** 2018-02-10

**Authors:** Jimena Soledad-Cadona, Ana Victoria Bustamante, Juliana González, Andrea Mariel-Sanso

**Affiliations:** Immunochemistry and Biotechnology Laboratory, Faculty of Veterinary Sciences, Tandil Veterinary Research Center, CONICET-CIC-UNCPBA, University Campus, Tandil 7000, Buenos Aires, Argentina; jcadona@vet.unicen.edu.ar (J.S.-C.); avbustaman@vet.unicen.edu.ar (A.V.B.); julianagonzalezrizz@gmail.com (J.G.)

**Keywords:** STEC, Pathogenicity islands, *nle* genes, seropathotypes

## Abstract

Shiga toxin-producing *Escherichia coli* (STEC) are foodborne pathogens associated with outbreaks and hemolytic-uremic syndrome. Cattle and meat foods are the main reservoir and infection source, respectively. Pathogenicity islands (PAIs) play an important role in STEC pathogenicity, and non-locus of the enterocyte effacement(LEE) effector (*nle*) genes present on them encode translocated substrates of the type III secretion system. A molecular risk assessment based on the evaluation of the *nle* content has been used to predict which STEC strains pose a risk to humans. The goal was to investigate the distribution of the PAIs OI (O-island)-36 *(nleB2*, *nleC*, *nleH1-1*, *nleD*), OI-57 (*nleG2-3*, *nleG5-2*, *nleG6-2*), OI-71 (*nleA*, *nleF*, *nleG*, *nleG2-1*, *nleG9*, *nleH1-2*) and OI-122 (*ent/espL2*, *nleB*, *nleE*, Z4321, Z4326, Z4332, Z4333) among 204 clinical, food and animal isolates belonging to 52 non-O157:H7 serotypes. Differences in the frequencies of genetic markers and a wide spectrum of PAI virulence profiles were found. In most LEE-negative strains, only module 1 (Z4321) of OI-122 was present. However, some unusual *eae*-negative strains were detected, which carried other PAI genes. The cluster analysis, excluding isolates that presented no genes, defined two major groups: *eae*-negative (determined as seropathotypes (SPTs) D, E or without determination, isolated from cattle or food) and *eae*-positive (mostly identified as SPTs B, C, or not determined).

## 1. Introduction

Shiga toxin-producing *Escherichia coli* (STEC) are heterogeneous foodborne pathogens associated with outbreaks and hemolytic-uremic syndrome (HUS) [1]. Cattle are the main reservoir of STEC and human infections are acquired mainly by ingesting food or water contaminated directly or indirectly with cattle feces. *E. coli* O157:H7 is the serotype most associated with diseases; however, more current studies have shown that the number of non-O157 STEC infections sometimes surpasses the number of STEC O157 infections [2].

Determinants of bacterial virulence are predominantly encoded by or associated with mobile genetic elements (MGEs) such as phages, plasmids, insertion elements, or transposons. A large number of these determinants are located within pathogenicity islands (PAIs), and can be exchanged among different bacterial species, and assembled and stabilized by selective pressure, leading to emerging pathogenic variants [3]. Increasing evidence shows that differences in virulence between pathogenic and non-pathogenic bacterial strains can be attributed in part to virulence genes located in PAIs [4,5].

Some pathogenic *Escherichia coli* use a type III secretion system (T3SS), encoded in the locus of the enterocyte effacement (LEE) pathogenicity island, to translocate a wide repertoire of effector proteins into the host cell in order to subvert cell signaling cascades and promote bacterial colonization and survival. Genes encoding type III-secreted effectors are located in the LEE and scattered throughout the chromosome. In addition to genes located on the LEE, a large number of non-LEE effector (*nle*) genes located on other PAIs have been identified in strains responsible for human infections. These genes are involved in various functions within the host cell, contributing to colonization and full virulence, including anti-apoptotic activities, disruption of host innate immune responses, increase of paracellular permeability, blockage of cell division, disruption of microtubule cytoskeleton, and inhibition of phagocytosis, among others [6].

A molecular risk assessment (MRA) approach based on the evaluation of the *nle* gene content has been used to predict which STEC strains pose a significant risk to human health [7,8,9,10]. Besides the production of Shiga toxins, the attaching and effacing phenotype encoded by the LEE and the presence of some non-LEE genomic O-islands (OI) such as OI-57, OI-71 and OI-122, are significantly associated with STEC types that are frequently involved in outbreaks and cause hemorrhagic colitis and HUS in humans [4,7,8,10].

OI-57 contains genes *nleG2-3*, *nleG6-2*, and *nleG5-2* in EDL933 [7,11]. NleG proteins are ubiquitin ligases. Although the exact functions of NleG2-3, NleG6-2, and NleG 5-2 are still unclear, similar proteins have been identified as effectors that suppress the immune response of the host [12].

OI-122 has, at least, six open reading frames (ORF), which show significant homology to known virulence genes. ORF Z4321 is homologous to *pagC* of *Salmonella enterica* serovar Typhimurium; ORF Z4326 is homologous to *sen* of *Shigella flexneri*; ORFs Z4328 and Z4329 are homologous to non-LEE effector (*nle*) genes *nleB* and *nleE* of *Citrobacter rodentium*; and ORFs Z4332 and Z4333 are homologous to the enterohemorrhagic *E. coli* (EHEC) factor for adherence gene cluster *efa1* and *efa2* found in STEC O157:H7 [4,8,13]. Wickham et al. [14] have described a modular arrangement of OI-122 genes based upon their association with each other across HUS-associated non-O157 STEC strains: module 1 contains Z4318, *pagC*, and Z4322; module 2 contains Z4323, *sen*, *nleB*, and *nleE*; and module 3 contains the *efa* gene cluster. The presence of putative transposases in OI-122 has led to the hypothesis that its elements are acquired or lost in a modular manner. It was shown that, while *pagC*, Z4322, *sen*, *nleB*, *nleE*, and *efa1* individually were more prevalent in non-O157 STEC associated with HUS, the simultaneous presence of all of these genes strengthened the association with serious disease [15].

Karmali et al. [4] proposed to group STEC strains into five seropathotypes (SPTs), from A through E, according to their reported frequencies in human illness, their known association with outbreaks and severe disease, and the presence of MGEs such as the LEE and the OI-122 PAI. While nearly all O157:H7/H- strains carry a complete OI-122 (COI-122), a progressive decrease in the prevalence of OI-122 genes in non-O157 STEC belonging to SPT B through E with a concomitant decreasing pathogenicity was observed.

Few studies to date have investigated PAIs other than LEE in STEC. Since PAIs may serve as useful markers to distinguish highly virulent strains from less-virulent strains, the aim of this study was to investigate the prevalence and the distribution of OI-36, OI-57, OI-71 and OI-122 among clinical, food and animal STEC isolates and evaluate the possible public health significance of these PAIs in STEC seropathotypes.

## 2. Materials and Methods 

### 2.1. Bacterial Strains

A total of 204 STEC isolates belonging to 52 non-O157:H7 serotypes were characterized in this study (see Table S1 in the Supplementary Material). All isolates are part of the Immunochemistry and Biotechnology Laboratory (FCV-UNCPBA, Argentina) collection and had been isolated in Argentina between 1998 and 2014. Twelve of the isolates were obtained from clinical cases (children up to six years old) (H), 73 from meat food, two from vegetables (F), and 117 from cattle (C). All isolates have been previously analyzed by polymerase chain reaction (PCR) for the presence of genes encoding for Shiga toxin 1 and 2 (*stx1* and *stx2*), intimin (*eae*), and enterohaemolysin (*ehxA*) [16,17,18,19,20]. Isolates were classified into SPTs, A to E, according to their serotypes, criteria described by Karmali et al. [4]. The assignment of SPTs was based on previously published information [4,5,21].

### 2.2. Detection of Non-Locus of the Enterocyte Effacement Effector Genes Encoded in Pathogenicity Islands

PCR was used to screen all isolates included in this study. They were examined by the presence of 16 markers of virulence effector genes encoded in genomic OIs different to LEE: OI-36 *(nleB2*, *nleC*, *nleH1-1*, *nleD*); OI-57 (*nleG2-3*, *nleG5-2*, *nleG6-2*); OI-71 (*nleA*, *nleF*, *nleG*, *nleG2-1*, *nleG9*, *nleH1-2*); and OI-122 (*ent/espL2*, *nleB*, *nleE*) (Table 1). The primers and PCR conditions for the amplification of non-LEE encoded effectors (*nle*) and *ent/espL2* were taken from Coombes et al. [7] and PCR products were visualized on a 1.5% or 2% agarose gel.

### 2.3. Detection of Particular Genes for the Presence of OI-122

All strains were screened for the presence of OI-122 by testing for four genes (Z4321, Z4326, Z4332, and Z4333) (Table 1). The primers and PCR conditions were taken from Karmali et al. [4]. PCR products were visualized on a 2% agarose gel.

According to Karmali´s proposal (*l.c*.), the presence of all four genes was considered the presence of a complete OI-122 (COI-122). The absence of one or more of the genes evidenced an incomplete OI-122, whereas the absence of all four genes indicated an absent OI-122.

### 2.4. Cluster Analysis

A cluster analysis of the studied isolates based on their PAI virulence-associated genes profiles was generated using the BioNumerics v.6.6 software (Applied Maths NV, Sint-Martens-Latem, Belgium).

## 3. Results

O-Islands are genetic regions absent from nonpathogenic *E. coli* and frequently contain virulence determinants. The distribution of 16 *nle* genes and four putative virulence genes encoded in four genomic PAIs (OI-36, OI-57, OI-71, OI-122) among non-O157 STEC strains were analyzed. 

**Figure 1:**
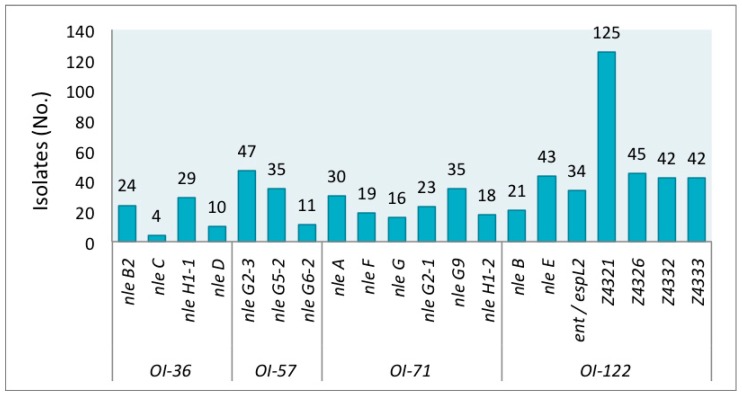
Prevalence of individual Pathogenicity Islands (PAIs) (OI-36, OI-57, OI-71, OI-122) virulence genes in shiga toxin-producing *Escherichia coli* (STEC) non-O157:H7.

The genetic markers were detected at different frequencies in the STEC studied group. The prevalence of individual virulence genes is shown in Figure 1. Among all strains, the gene Z4321 (*pagC*) encoded in OI-122 was the most prevalent, resulting in both LEE-positive and LEE-negative isolates. In contrast, *nleC* encoded on OI-36 was the least prevalent, found in only four isolates of O26:H11 (from human), O145:NM (2 from humans), and O165:NM (from cattle).

**Figure 2:**
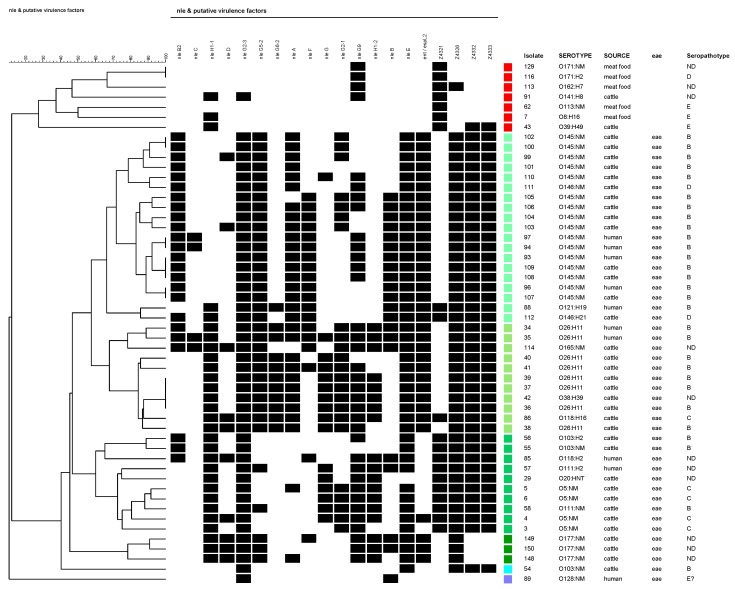
Cluster analysis of PAIs OI-36, OI-57, OI-71, and OI-122 virulence-associated genes in clinical, cattle, meat food, and vegetable non-O157 STEC isolates. A black box indicates the presence of the target sequence in the strain and a white box, the absence. Isolates that presented no gene were excluded. The colour red represents all the *eae*-negative isolates; tree branch corresponding to isolate #62 also includes all the isolates with profile Z4321-positive, O2:H5 (1), O2:NM (1), O8:H16 (2), O15:H21 (1), O20:H19 (1), O22:H8 (3), O39:H49 (2), **O88:H21** (1), **O91:H21** (5), **O113:NM** (10: strain #62 + 9), O113:H21 (5), **O116:H21** (1), O117:H7 (6), **O171:HNT**, O171:H2 (9), O171:NM (1), **O174:H21** (14), O178:H19 (4), **O185:H7** (1), **ONT:H7** (5), **ONT:H8** (2), ONT:H21 (13), ONT:HNT (1), and **ONT:NM** (16). All of them were isolated from cattle or meat food. All the studied strains belonging to serotypes in bold had the same profile. The remaining colours are *eae*-positive isolates.

A group of forty-six isolates presented no gene. Forty-four strains (from cattle, meat food, and vegetables) out of these 46 strains were *eae*-negative, as expected, but, strikingly, two were *eae*-positive. Both were O145:NM strains isolated from humans. The serotypes with absence of the genes studied were: O8:H19, O20:H7, O20:H19, O25:H19, O79:H19, O117:H2, O120:H19, O141:H7, O175:H8, and ONT:H19. A second group comprised of 106 isolates carried only Z4321 (Table S1). Serotypes of these Z4321-positive strains are detailed in the legend of Figure 2.

Most *eae*-negative strains lacked all 20 tested virulence genes; however, six exceptional strains, which carried additional genes to Z4321 were detected: *nleH1-1*, *nleG2-3*, *nleG9*, Z4326, Z4332, and Z4333. Gene *nleH1-1* was detected in strains that belonged to serotypes O8:H16 (#7) and O39:H49 (#43, which also presented Z4332 and Z4333); *nleG9* in strains which belonged to serotypes O141:H8 (#91), O171:H2 (#116), and O171:NM (#129); and *nleH1-1* + *nleG2-3* + *nleG9* in one O162:H7 (#113) strain (Figure 2).

The O26:H11 strains are among the strains that have the largest number of virulence genes. They showed 14 to 18 of the 20 genes. Those with more detected genes (16 and 18) were from humans. The strain with 18 genes only lacked *nleD* and *Z4321* (OI-57 and 71 were complete). Notably, two cattle isolates belonging to O118:H16 and O165:NM carried 16 of the 20 analyzed genes, with the presence of genes of each OI.

Table 2 gives details on the prevalence of PAI genes in five STEC *eae*-positive serotypes represented by more than one isolate. Depending on the serotype, some *nle* genes were never detected, for example, *nleB2*, *nleC*, *nleG5-2*, *nleG6-2*, *nleF*, and *nleB* in O5:NM or *nleH1-1*, *nleG6-2*, *nleH1-2*, and *Z4321* in O145:NM.

In several serotypes (O5:NM, O8:H16, O20:H19, O26:H11, O103:NM, O117:H7, O141:H8, O145:NM, O171:H2, O171:NM, O174:H21, O177:NM, O178:H19) more than one virulence profile were found.

**Figure 3:**
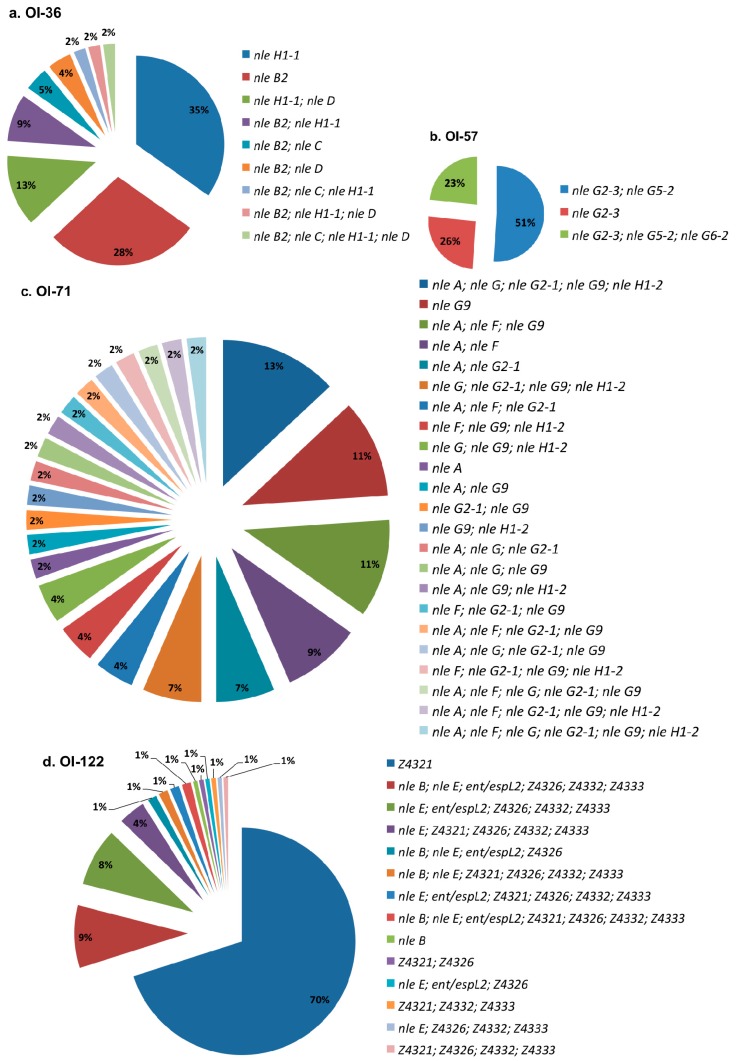
Virulence profiles distribution for each studied pathogenicity island in OI-encoded genes-positive STEC non-O157:H7. (**a**) OI-36; (**b**) OI-57; (**c**) OI-71; (**d**) OI-122.

Figure 3 shows the distribution of profiles for each investigated PAI. Among the analyzed STEC, (a) OI-36 showed nine profiles, with the most prevalent being *nleH1-1* and *nleB2*; (b) OI-57 exhibited three profiles, including each one of these profiles with one, two, or three genes. Approximately half of the strains presented *nleG2-3* and *nleG5-2* simultaneously; (c) OI-71 showed 23 profiles, with each one represented by 2–13% of the strains; and (d) OI-122 displayed 14 profiles, with the most prevalent, Z4321, present in 70% of the isolates.

Only O26:H11 (humans -H- and cattle -C-), O38:H39 (C), O118:H16 (C), and O121:H19 (H) strains carried a complete OI-57. Meanwhile, only one O165:NM strain isolated from cattle had a complete OI-36 and one other, O26:H11 (H), presented a complete OI-71 and only three strains, one O111:H2 and one O121:H19 from a human, and one O146:H21 from cattle carried a complete OI-122. All of them were *eae*-positive serotypes.

**Figure 4:**
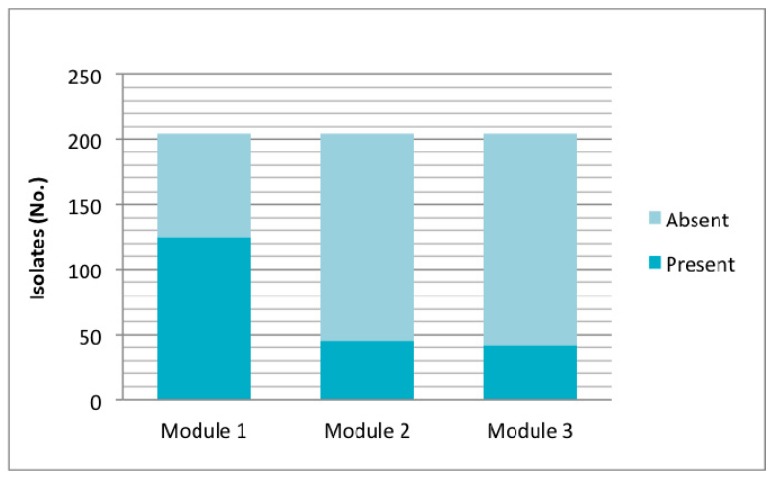
Presence of OI-122 according to marker genes located in the three modules; module 1: Z4321; module 2: *nleB*, *nleE*, *ent/espL2*, Z4326; and module 3: Z4332, Z4333. Presence of each module was considered by detecting at least one marker of it.

Particularly, in relation to OI-122, its presence was determined by seven marker genes located in different regions of the island. Of the 204 isolates studied, 157 (76.9%) were positive for at least one gene of OI-122, of which 45 isolates were *eae*+ and 112 *eae*−. Among the *eae*-isolates, three profiles were obtained: Z4321 (*n* = 110) (module 1); Z4321/Z4326 (*n* = 1) (modules 1 and 2); and Z4321/Z4332/Z4333 (modules 1 and 3) (*n* = 1) (Figure 4). Analyzing OI-122 in a modular way, module 1 was present (at least presence of one gene) in 61.3% of the strains; module 2 was present in 22.5%; and module 3 was present in 20.5% (Figure 4).

According to the criteria described by Karmali et al. [4] based on the detection of COI-122 that requires testing for four virulence putative genes (Z4321; Z4326; Z4332; Z4333), 13 of 204 (6.4%) isolates studied had a complete OI-122, 143 (70.1%) had an incomplete OI-122, and OI-122 was absent in 48 (23.5%) strains.

The cluster analysis (which did not include isolates that presented no genes), presented in Figure 2, divided the studied set of strains into two major groups: (A) *eae*-negative, and (B) *eae*-positive isolates. Within the *eae*-negative group, the isolates were determined as belonging to SPTs D, E, or without determination and were isolated from cattle or meat food. Within the *eae*-positive group, the isolates were mostly identified as SPTs B, C, or not determined. Exceptionally, two isolates were characterized as SPT D (O146:H21 and O146:NM, both from cattle). Among this group, four principal branches could be differentiated (represented in four different green shades in Figure 2): (B1) O145:NM (+O121:H8+O146:H21/NM) isolates; (B2) O26:H11 (+O118:H16+O165:NM) isolates; (B3) O5, O103, O111 (+O20:HNT+O118:H2) isolates; and (B4) O177:NM isolates.

## 4. Discussion

The presence of *nle* genes and the number of genes carried by an *E. coli* strain are important criteria for estimating its virulence potential [7]. Previous evidence shows that virulence genes located on PAIs can be used to identify new and emerging pathogenic bacteria [4,5]. In this study, 18 isolates carried more than 60% of the 20 OI-genes analyzed. These isolates belong to the O5:NM, O26:H11, O38:H39, O118:H2/H16, O121:H19, O145:NM, O146:H21, and O165:NM serotypes. From these, four isolates, belonging to O26:H11 from humans, and O118:H16 and O165:NM from cattle, were classified as “virulence top ranking”, presenting between 80 and 90% of the genes. The O26:H11 strains represent a group with a high virulence potential for humans. This is also corroborated by their serotype, which is associated with the classical EHEC serotype. 

This study detected several profiles for each PAI, especially in different serotypes, and that one or more PAIs virulence genes could be absent in STEC non-O157. In agreement with Ju et al. [5,23], we postulate that PAI could be unstable in STEC. However, as highlighted in the results summarized in Table 2, the presence or absence of particular genes´ intra-serotype is not random; instead, there are specific arrays of virulence factors. Further studies using whole-genome sequencing might identify additional virulence markers and increase the understanding of their contribution to human disease.

In spite of the fact that LEE is important, it is not essential for STEC pathogenesis, and sporadic cases and small outbreaks of STEC infections have been caused by LEE-negative strains [24]. Although the absence of *eae* was associated with the absence of many of the virulence genes, unlike previous publications, such as Franz et al. [22], this study detected some unusual *eae*-negative cattle and food strains belonging to serotypes O8:H16, O39:H49, O141:H8, O162:H7, and O171:H2/NM, which carried genes *nleH1-1*, *nleG2-3*, *nleG9*, Z4326, Z4332, or Z4333. Such strains, unlike other LEE-negative strains, may cause human diseases. Indeed, some of these serotypes, for example, O171:H2 and O171:NM, have been associated with HUS and/or bloody diarrhea in children in Argentina [25]. Therefore, the risk represented by them to public health should be monitored. On the contrary, two *eae*-positive strains, O145:NM isolated from a human, lacked all of the studied PAI genes.

It has been proposed and agreed that COI-122–positive strains belong to SPTs associated with outbreaks (A and B) and SPTs associated with HUS (A, B, and C) [4,14,15]. However, in this study, some COI-122–positive strains identified with different SPTs or not determined, O20:HNT (SPT not determined), O146:H21 (D) strains from cattle, and O111:H2 and O118:H2 (SPT not determined) from humans were detected. Also, in relation to OI-122, and coincidently with the results from Konczy et al. [8], results showed that when LEE was absent, only module 1 (gene Z4321) was present. These strains that carry the *pagC*-like gene exclusively (modules 2 and 3 absent) would have a non-synonymous substitution in (His→Gln) [8]. Wickham et al. [14] postulated that there was a significant association between the presence of a combination of OI-122 markers, such as the *pagC*-like gene and *sen*, *nleB*, and *efa-1*, and HUS after infection in non-O157 *E. coli*. Four strains with this profile: three human strains (one O111:H2, one O118:H2, and one O121:H19), and, interestingly, one O146:H21 strain isolated from the bovine group were found. 

Newton et al. [26] proposed that NleE and NleB (OI-122) contribute to pathogenesis by inhibiting an initial host inflammatory response (contribute to the suppression of innate signaling pathways) to allow the bacteria to persist in the early stages of infection. NleE appears to obstruct nuclear translocation of Rel family transcriptional activators, while allowing the nuclear import of a transcriptional repressor, resulting in the suppression of IL8 expression. NleB prevents translocation of the immune regulator nuclear factor kappa-light-chain-enhancer of activated B cells (NF-kB) to the cell nucleus [26]. Gene *nleB* was detected in O26:H11, O111:H2, O118:H2, O121:H19, O128:NM, and O145:NM strains isolated from patients, and in O145:NM, O146:H21, O165:NM, and O177:NM cattle strains. The O26:H11 strains from humans presented *nleB* and *nleE*, but cattle O26:H11 strains presented only *nleE*. Unlike a study of Karmali et al. [4] in which they postulated that in cases where a serotype was represented by more than one strain, all strains belonging to the same serotype had identical patterns of distribution of OI-122 genes, in this work different virulence profiles for OI-122 in isolates belonging to the same serotype were found (see Figure 2). In previous work, in which a subset of these strains was analyzed using multilocus sequence typing (MLST), different sequence types (STs) in the same serotype were detected [27]. Further studies should determine if different STs correspond to different PAI-virulence profiles.

According to the results from Ju et al. [5], some of OI-122 and OI-57 *nle* (*nleB*, *nleG2-3*, *nleG5-2*, and *nleG6-2*) were primarily associated with *eae*-positive STEC strains and associated with SPTs that cause severe diseases (SPT A, B, C), offering an important basis for STEC MRA. But, based on the MRA framework, which uses *nle* genes as sole markers, all *eae*-negative virulence STEC strains, including HUS-associated O113:H21 and O91:H21, would be categorized as harmless. Therefore, we agree that additional markers, especially for *eae*-negative STEC, are needed to absolutely predict the potential of an STEC strain to cause disease. Recently, a novel PAI named Locus of Adhesion and Autoaggregation (LAA), exclusively present in a subset of LEE-negative STEC strains, with the mostly clinically relevant ones including O91:H21, O113:H21, and O174:H21, has been proposed. Several virulence factors, among them Hes, which participates in colonization-associated phenotypes, and other ones participating in adhesion and autoaggregation such as Pag-C, Iha, and, Agn43 are encoded in it [28].

## Figures and Tables

**Table 1:** Virulence genes encoded in pathogenicity islands (PAIs) analyzed in this study.

PAIs	Target	Encoded Protein or Family Effector ^1^
O-Island 36	*nle B2*	Non-LEE encoded type III effector
	*nle C*	Immunomodulation, zinc-metalloprotease
	*nle H1-1*	Immunomodulation
	*nle D*	Immunomodulation, zinc-metalloprotease
O-Island 57	*nle G2-3*	Ubiquitin ligase
	*nle G5-2*	Ubiquitin ligase
	*nle G6-2*	Ubiquitin ligase
O-Island 71	*nle A*	Disruption tight junctions and protein trafficking
	*nle F*	Disruption protein trafficking
	*nle G*	Ubiquitin ligase
	*nle G2-1*	Ubiquitin ligase
	*nle G9*	Ubiquitin ligase
	*nle H1-2*	Immunomodulation
O-Island 122	*nle B*	Immunomodulation
	*nle E*	Immunomodulation
	*ent/espL2*	Microcolony formation and F-actin aggregation
	Z4321 (*pagC*)	Similarity to *Salmonella enterica* serovar Typhimurium PhoP-activated gene C
	Z4326 (*sen*)	Similarity to *Shigella flexneri* enterotoxin 2
	Z4332 (*efa1*)	EHEC factor for adherence
	Z4333 (*efa2*)	EHEC factor for adherence

^1^ From Karmali et al. [4], Coombes et al. [7], Konczy et al. [8] and Franz et al. [22]; EHEC: Enterohemorrhagic *E. coli*, LEE: Locus of the enterocyte effacement.

**Table 2:** Prevalence of PAI virulence genes in STEC *eae*-positive serotypes represented by more than one isolate: O5:NM, O26:H11, O103 (O103:NM and O103:H2), O145:NM, and O177:NM.

		% Isolates Positive for Gene				
PAI	Gene	O5:NM (*n = 4*)	O26:H11 (*n = 8*)	O103 (*n = 3*)	O145:NM (*n = 18*)	O177:NM (*n = 3*)
O-Island 36	*nle B2*	0	25	66.7	88.9	0
	*nle C*	0	12.5	0	11.1	0
	*nle H1-1*	100	100	66.7	0	100
	*nle D*	25	12.5	0	11.1	100
O-Island 57	*nle G2-3*	100	100	100	94.4	100
	*nle G5-2*	0	100	0	88.9	100
	*nle G6-2*	0	100	0	0	0
O-Island 71	*nle A*	25	100	0	83.3	33.3
	*nle F*	0	37.5	0	61.1	33.3
	*nle G*	75	87.5	0	5.5	0
	*nle G2-1*	100	87.5	0	38.9	0
	*nle G9*	100	87.5	33.3	44.4	100
	*nle H1-2*	75	62.5	0	0	100
O-Island 122	*nle B*	0	25	0	61.1	66.7
	*nle E*	100	100	100	88.9	100
	*ent/espL2*	25	100	0	88.9	100
	*Z4321* (*pagC*)	100	0	66.7	0	0
	*Z4326* (*sen*)	100	100	100	88.9	100
	*Z4332* (*efa1*)	100	100	100	88.9	0
	*Z4333* (*efa2*)	100	100	100	88.9	0

## Supplementary Materials

The following are available online at http://www.mdpi.com/2073-4425/9/2/81/s1. Table S1: Non-O157 STEC isolates analyzed in this study and PAIs-encoded virulence genes results.
