# Erratum: Jimena Soledad Cadona, et al.; Pathogenicity Islands Distribution in Non-O157 Shiga Toxin-Producing *Escherichia coli* (STEC). *Genes* 2018, *9*, 81

**DOI:** 10.3390/genes9040222

**Published:** 2018-04-20

**Authors:** Jimena Soledad Cadona, Ana Victoria Bustamante, Juliana González, Andrea Mariel Sanso

**Affiliations:** Immunochemistry and Biotechnology Laboratory, Faculty of Veterinary Sciences, Tandil Veterinary Research Center, CONICET-CIC-UNCPBA, University Campus, Tandil 7000, Argentina; jcadona@vet.unicen.edu.ar (J.S.C.); avbustaman@vet.unicen.edu.ar (A.V.B.); julianagonzalezrizz@gmail.com (J.G.)

We wish to make the following correction to the paper by Soledad-Cadona et al. [1]. The name and surname of two authors should be correctly written as Jimena Soledad Cadona (J.S.C.) instead of Jimena Soledad-Cadona (J.S.-C.) and Andrea Mariel Sanso (A.M.S.) instead of Andrea Mariel-Sanso (A.M.-S). Cadona and Sanso are their only surnames. With this modification, the authors’ work will be properly listed by search engines. 

The new version of the reference is:

Cadona, J.S.; Bustamante, A.V.; González, J.; Sanso, A.M. Pathogenicity Islands Distribution in Non-O157 Shiga Toxin-Producing *Escherichia coli* (STEC). *Genes* **2018**, *9*, 81.

We apologize for any trouble this might have made for you. The change does not affect the scientific results. The manuscript will be updated, and the original will remain online on the article webpage.
